# Influence of number of individuals and observations per individual on a model of community structure

**DOI:** 10.1371/journal.pone.0252471

**Published:** 2021-06-17

**Authors:** Julia Sunga, Quinn M. R. Webber, Hugh G. Broders

**Affiliations:** 1 Department of Biology, University of Waterloo, Waterloo, Ontario, Canada; 2 Cognitive and Behavioural Ecology Interdisciplinary Program, Memorial University of Newfoundland, St. John’s, Newfoundland and Labrador, Canada; 3 Department of Biology, Saint Mary’s University, Halifax, Nova Scotia, Canada; Institut de Recherche pour le Developpement, FRANCE

## Abstract

Social network analysis is increasingly applied to understand animal groups. However, it is rarely feasible to observe every interaction among all individuals in natural populations. Studies have assessed how missing information affects estimates of individual network positions, but less attention has been paid to metrics that characterize overall network structure such as modularity, clustering coefficient, and density. In cases such as groups displaying fission-fusion dynamics, where subgroups break apart and rejoin in changing conformations, missing information may affect estimates of global network structure differently than in groups with distinctly separated communities due to the influence single individuals can have on the connectivity of the network. Using a bat maternity group showing fission-fusion dynamics, we quantify the effect of missing data on global network measures including community detection. In our system, estimating the number of communities was less reliable than detecting community structure. Further, reliably assorting individual bats into communities required fewer individuals and fewer observations per individual than to estimate the number of communities. Specifically, our metrics of global network structure (i.e., graph density, clustering coefficient, R_com_) approached the ‘real’ values with increasing numbers of observations per individual and, as the number of individuals included increased, the variance in these estimates decreased. Similar to previous studies, we recommend that more observations per individual should be prioritized over including more individuals when resources are limited. We recommend caution when making conclusions about animal social networks when a substantial number of individuals or observations are missing, and when possible, suggest subsampling large datasets to observe how estimates are influenced by sampling intensity. Our study serves as an example of the reliability, or lack thereof, of global network measures with missing information, but further work is needed to determine how estimates will vary with different data collection methods, network structures, and sampling periods.

## Introduction

Social network analysis permits an in-depth understanding of population-level social structure and has been widely applied to study the organization of animal groups [1–3]. Social groups may vary in size, composition, and structure, and large groups may show hierarchical organization, with higher order groups comprised of subgroups [4,5]. These subgroups are not always static in structure, demonstrating fission-fusion dynamics, as they merge and split through space and time, and consistencies in subgroup composition may result in the delineation of social communities [6–8]. Social communities, hereafter ‘communities’, are defined by the appearance of social preference among discrete clusters of individuals that associate more strongly with members of the same community than with members of other communities [9]. For fission-fusion societies, individuals can interact to varying extents with conspecifics from multiple social communities making the identification of community boundaries difficult. Community structure can still be delineated, when sufficient data exists, based on repeated or consistent subgroup composition and associations among individuals. An understanding of the degree to which associations between individuals persist, and the locations where associations occur, can provide insight into whether community formation is the result of passive processes such as use of a common resource, or active processes such as social preference among conspecifics, among other factors [1,2,10–12]. Testing hypotheses about social structure in groups with fission-fusion dynamics requires sufficient data on interactions among individuals to reliably assign community membership [3].

A common challenge for studies using social network analysis in natural systems is collecting enough data to reliably estimate elements of the group’s social structure, particularly as they relate to social communities. Specifically, the proportion of a group, or total number of individuals sampled, and the number of observations per individual must be sufficient to reflect the dynamics of the true network. In a recent review of social network literature, Webber & Vander Wal [3] identified the median number of individuals included per network was 15 (range 4–1406), but many studies did not indicate what proportion of the overall population this may represent. Further, the minimum number of observations included per individual varies widely among studies and can have important effects on network estimates. For example, based on assessment of individual network metric performance, Farine and Strandburg-Peshkin [13] recommended that there should be at least 20 observational periods where a pair of individuals is observed either together or apart [see also 8,14–17]. Other studies select a median number of observations per individual as a decision rule [i.e., 9], which, in well sampled populations, may unnecessarily omit individuals or pairs of individuals that have fewer observations than the designated threshold. In contrast, in poorly sampled populations, the median number of observations per individual may retain individuals that do not have sufficient information with which to estimate their network position. Generally, increasing the minimum number of observations required per individual reduces the number of individuals that can be included in an analysis, effectively decreasing the estimated network size and increasing the proportion of the population that is analytically considered as ‘missing’ [18]. Multiple studies have assessed the effect of missing data on the estimation of animal social networks, largely focusing on impacts on estimates of an individual’s position in the network (e.g., degree, centrality) [14,15,17,19]. Specific thresholds can vary based on the network metrics of interest and the type of network, but important broad patterns relating to the effect of missing data are consistent across most studies. Across all network sizes, studies have suggested minimums of 10–50% of the population sampled and unsurprisingly, the more observations that can be obtained of these individuals, the better [14,17]. However, Davis et al. [20] also note that in some systems, increased sampling may not be a priority because at some point networks become ‘saturated’ such that further sampling does not markedly increase estimate accuracy unless all individuals or interactions can be observed, a nearly impossible feat in many animal groups. Importantly, requirements of a minimum number of observations per dyad or median threshold should vary with the type and timing of the social system being studied [19], the behaviour or interaction of interest and the method of sampling that is applied [20]. Thus, recommendations may vary among different social systems (e.g., those displaying fission-fusion or multilevel dynamics). It has also been recommended to more intensely sample a smaller proportion of the population when resources are limited [14,17]. However, for fission-fusion societies, the potential for ephemeral social connections, which can impact the functions and consequences of animal societies (e.g., information transfer and disease transmission [21]), differs from societies with more stable community composition. Meanwhile, too few observations per individual may lead to spurious estimates of the strength of connections between individuals and approximations of network structure. Further work is needed to determine the suitability of these recommendations across different types of animal groups. We therefore expect a need to balance minimum requirements for observations to accurately assess individual connections, while also including as many individuals as possible to model a larger proportion of the overall population.

When investigating a new system, various strategies can be applied to assess the robustness of estimates to missing data, and to determine what amount of data is needed for a desired level of confidence. Bootstrapping is the simplest method to apply, where aspects of the network are repeatedly assessed to derive multiple estimates of a statistic to characterize its distribution [14,22]. This technique, particularly that of resampling with varying numbers of samples included, is integral to the community assortativity estimate process described by Shizuka and Farine [22]and has also been applied to assess the robustness of network measures [14,17]. Further, a variation of bootstrapping, jackknifing, in which data is systematically removed, is commonly used to assess the error associated with estimates of lagged associate rates [8]. Bayesian methods can be applied to assess the uncertainty around edge weight estimates [13].

Here, we use bootstrapping to examine the impact of varying number of observations per individual and number of individuals included in social network analysis on estimates of global network structure, particularly as they relate to the presence of communities, in a group displaying fission-fusion dynamics. Generally, it is expected that sample size is inversely correlated with the discrepancy between the true network structure and estimates of it, but that reasonable estimates can be obtained with missing data. Using an empirical dataset of a large group of little brown myotis (*Myotis lucifugus*) displaying fission-fusion dynamics, we quantified the impacts of two distinct sampling considerations on estimates of global network metrics and community structure. Specifically, we assessed how variation in each of the number of individuals and number of observations per individual affected the discrepancy of the sub-sampled network in estimates of graph density, clustering coefficient, the number of communities, and the assortment of individuals into communities.

## Materials and methods

### Sample population

We used data from a long-term study of wild little brown myotis roosting in artificial structures at Salmonier Nature Park, Newfoundland, Canada in 2016. Individuals were implanted with passive integrated transponder (PIT) tags and readers (Trovan, Ltd. United Kingdom) were deployed at 11 roost boxes within a ≈1 km^2^ area of the park, each of which were regularly used throughout the study period. Other natural and anthropogenic roosts existed in the area that were not monitored. Readers recorded observations of when individuals entered and exited roost boxes and the last observation before sunrise constituted the assigned daytime roost locations of individual bats. Dyadic associations were based on the gambit-of-the-group assumption [8], such that individuals were assumed to associate if they roosted in the same box on the same day. This assumption in commonly used in studies of bat roosting behaviours [examples include 23–25]. It is known that there were missing observations and that at least 50% of individuals in the population were not tagged nor included in this study. Although it may, this dataset is not intended to represent the true population as characterization of the actual true population is not the purpose of this study. Rather, our aim is to quantify differences in estimates between samples where all available information is included, to those with only partial data.

All animal handling protocol was approved by the animal care committee of Saint Mary’s University, Halifax, Nova Scotia (AUP #16–12). Wildlife scientific research permits were also obtained from the Government of Newfoundland and Labrador, Department of Fisheries and Land Resources, Forestry and Wildlife Branch (# WLR2016-12).

### Generating the ‘observed’ network

All detections were filtered for adult female bats that were each observed on ≥ 40 days, which resulted in 99 individuals. For each individual, 40 of their observations (day records of an individual bat in a particular roost) were randomly selected to generate a balanced dataset (all individuals observed an equal number of times, albeit not on the same days) from which we calculated an ‘observed’ social network that spanned 131 observation days. These numbers were selected to provide more observations per individual than has been assessed in other studies, e.g., Franks et al. [14] which assessed reliability up to a maximum of 20 censuses, while still allowing multiple levels of sub-setting of the number of individuals included. Although a network with a maximum of 40 observations removes additional information on individuals in an already incomplete dataset, we used this threshold to remove any impact of individuals with uneven numbers of available observations. Further, the ‘observed’ network was not assumed to be a reliable estimate of the true underlying population, and as such, all findings and implications focus on discrepancies between metrics of the observed network with estimates based on sub-sampled networks.

From our data, we calculated an observed network based on the instances of co-roosting, which represent individuals observed in the same roost on the same day. We first calculated the Half Weight Index (HWI) [8] between all dyads based on the proportion of days spent roosting together or apart using package “asnipe” [26]. The HWI represented edges in the network. We selected HWI, as opposed to the simple ratio index (SRI), as our association index of choice based on the sampling regime of our study. Although SRI is being increasingly recommended as an association index, the HWI accounts for missing individuals within a given sampling period [8]. In this system, it is likely there are a number of unmonitored roosts within the study area and given we allowed for a maximum of 40 observations out of a possible 131 nights on which observations were possible, it is reasonable to expect there are many days when only one bat in a given dyad is detected. Based on our observed network, we calculated three global social network metrics. First, we calculated modularity (Q), which is a measure of the number of edges within communities compared to the expected number if all edges were placed randomly [5]. Based on network modularity, we estimated the number of communities using the “igraph” package *cluster_fast_greedy* community detection algorithm [27], a process which iteratively divides the network until the ratio of within to among community connections peaks. Second we calculated the clustering coefficient (function: *transitivity*), defined as the proportion of triads that have three edges compared to the number of triads with two edges [28]. Third, we calculated graph density (function: *edge_density*), defined as the number of observed edges across the whole network divided by the number of possible edges [28]. Both graph density and clustering coefficient were calculated using the R package “igraph” [27].

We then assessed evidence for the division of the network into communities. We generated 1000 random networks using “spatsoc” [29] to randomly swap individuals among roosting subgroups on a given day, while maintaining the number of available roosting boxes and individuals constant within days, generating distributions of Q, clustering coefficient, and graph density values. These parameters were held constant to create null model networks that reasonably approximated possible network structure if all roosting decisions were random. A p-value was calculated, via Monte Carlo methods, based on the percent of random network Q values that fell above that of the original ‘observed’ network, where a p-value of < 0.05 indicated significant evidence for community structure. This measure may only support an inference that there is evidence for community structure and does not provide confidence in the estimated number of communities or the assignment of individuals to these communities. Next, using a metric proposed by Shizuka and Farine [22], we examined the confidence in the assignment of individuals to communities by calculating a community assortativity coefficient (R_com_). The R_com_ method uses bootstrapping of the ‘observed’ network to calculate consistency in individual community assignment, resulting in a network-level value between -1 and 1. Values closer to 1 indicate high confidence in community assortment while values closer to 0 indicate little confidence in community assortment. Shizuka and Farine [22] recommend a threshold of 0.5 for reasonable confidence in community assortment. We used 1000 bootstraps to calculate R_com_ and this process was repeated 10 times to obtain an average value for the ‘observed’ network.

### Sampled networks

In this study, we assessed the influence of two distinct sampling considerations, the number of individuals included, and the number of observations included per individual. All sample networks were based on randomly selecting a subset of individuals and observations from the above generated ‘observed’ network. Individuals were randomly sampled to generate subsets of 5, 10, 15, 20, 25, 35, 50, 75, and 99 (all) individuals. Within each of these network sizes, we randomly selected 1, 5, 15, 10, 20, 30, or 40 (all available from ‘observed’ network) observations for each individual to generate balanced sample networks. This sampling process was repeated 100 times for each combination of number of individuals and number of observations per individual. A sample network of 99 individuals with 40 observations per individual was not created as this would represent the complete ‘observed’ network.

For all sample networks, the number of subgroups and Q were estimated as above for the ‘observed’ network. P-values of Q were calculated by generating 1000 random networks for each sample network and comparing the distribution of Q values via Monte Carlo methods as above. These random networks shuffled the roost in which an individual bat roosted on a particular day, and were generated with “spatsoc” as above [29]. Those with a p-value < 0.05, indicating significant evidence for subgroups structure were assigned a YES (1), while those with a p-value > 0.05 were assigned a NO (0). In all sample networks, we also estimated network graph density and clustering coefficient as for the ‘observed’ network.

For each of the 100 sample networks related to each sampling regime, we assessed the accuracy in assignment of dyads to communities compared to the observed. Specifically, we generated individual-by-individual matrices, each of which indicated whether a dyad was estimated to occupy the same or different communities and whether this assignment was consistent with the observed network. We then generated a proportional measure of similarity. The similarity measure was a proportion that ranged from 0 to 1, where 0 indicated that no dyads were assigned to communities correctly based on the ‘observed’ network, and 1 indicated that all dyads were correctly assigned to communities compared to the ‘observed’ network.

Finally, R_com_ values were also calculated as outlined by Shizuka and Farine [22]. The number of bootstraps was set to 1000, regardless of the network size. Although this may be redundant in smaller networks, this ensured sufficient subsampling for all sample networks. On occasion, particularly in networks with only 1 observation per individual, no connections between individuals were present in the sample network, resulting in a Q value of zero and returning a network where the number of detected communities was equal to the number of individuals present. This scenario does not constitute community structure and because there was no ability to assign individuals to communities the community logistic value was coded as a NO (0) while R_com_ values were set to 0.

### Statistical analyses

To assess how sampling regime affected estimates of community structure, the absolute value of the difference in estimated number of communities between the ‘observed’ network and the sample networks was calculated. Multiple linear regression was used to assess whether the independent variables of the number of observations, the number of individuals, and the interaction between these factors influenced the difference between the sample and ‘observed’ network in estimated number of communities. Both response and independent variables were log-transformed to meet model assumptions of heteroscedasticity of residuals. We also applied a multiple linear regression to assess the influence of these factors on the proportion of similarity of dyadic community assignment between sample networks and the ‘observed’ network.

For all sample networks, we calculated the absolute difference from the ‘observed’ network for measures of graph density and clustering coefficient. Here, we also applied multiple linear regressions of the number of individuals, the number of observations per individual, and the interaction of these terms to assess their impact on these metric estimates. For our assessment of graph density, both independent and response variables were log transformed to meet assumptions of homoscedasticity of residuals. The transformation was not required for the analysis of clustering coefficient.

Often, network analysis simply aims to determine whether there is sufficient evidence of community structure in a study population [e.g. 24]. A logistic regression was performed on whether there was evidence for community structure (p < 0.05) in the sample networks as there was in the ‘observed’ network. Number of individuals, number of observations per individual, and their interaction were used as the independent variables and the response variable was whether there was significant evidence for community structure (1), or insufficient evidence (0; p > 0.05).

We also tested how different sampling regimes affected the ability to confidently assort individuals based on community assortativity (R_com_ > 0.5; Shizuka & Farine, 2016). Other assortativity measures, measures of how strongly nodes associate with those most like themselves, have been reported to be artificially high in poorly sampled systems [14]. Thus, a linearly increasing R_com_ value was not expected with the addition of more information. We therefore tested the probability of obtaining a R_com_ estimate that indicated confidence in individual assortment (R_com_ ≥ 0.5), without overestimating this confidence by exceeding the value of the ‘observed’ network. Values within this range were coded as acceptable (1) while those above or below this range as unacceptable (0). A logistic regression was then performed with this binary response variable and the number of observations, number of individuals, and the interaction between these factors as independent variables. All sampling, randomizations, network metric calculation, and statistical analysis were performed in R version 4.0.0 [30].

## Results

### Relationship between sampling regime and global network measures

**Fig 1:**
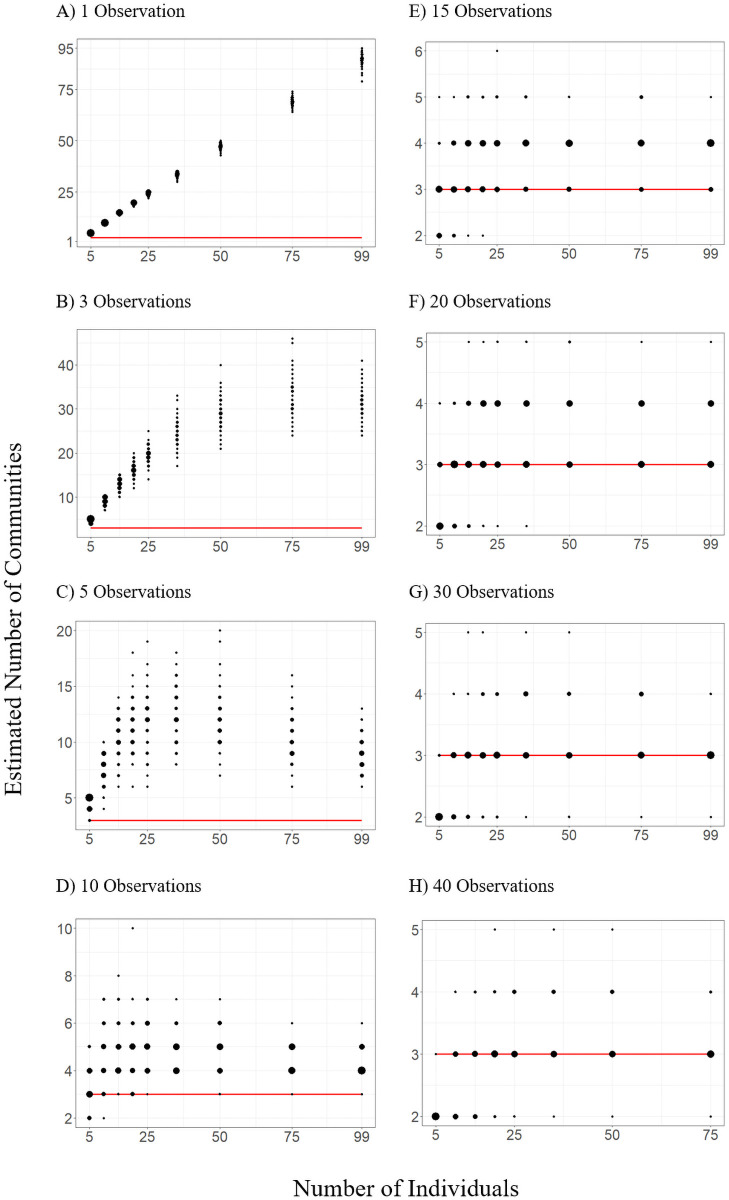
Variation in the estimated number of communities under various scenarios of number of individuals sampled and the number of observations per individual (panels A-H). Point sizes are scaled based on the number of times a value occurred. The red horizontal line represents the actual number of communities in the ‘observed’ population from which all other scenarios were sampled (subgroups = 3). Note, the changing y-axis scale among panels.

The ‘observed’ network of 99 individuals with 40 observations per individual was estimated, with significant support to have community structure (Q = 0.285, p < 0.0001) with 3 communities estimated. The ‘observed’ network also had a graph density of 0.675, a clustering coefficient of 0.828 and a R_com_ of 0.858, indicating a high-level of confidence in the assortment of individuals into communities. Across all sample networks, the estimated number of communities ranged from 2 to 95 (median = 4, SE ± 0.177). Multiple linear regressions revealed that each of the number of individuals (β = 1.069, SE = 0.012, p < 0.001), number of observations per individual (β = 0.297, SE = 0.0166, p < 0.001), and the interaction between these terms (β = -0.368, SE = 0.005, p < 0.001) explained discrepancy between the estimated number of communities in the ‘observed’ network and the number estimated by sample networks (p < 0.001, R^2^ = 0.871). Generally, error associated with our estimates was large (range 0–92, SE = 0.376) when the number of observations was low (< 10) and increased when more individuals were included with few (< 10) observations (Fig 1). Regardless of the number of individuals included the number of communities was consistently estimated to be between 2 to 4 (within one of the ‘observed’ network) when at least 15 observations per individual (96.4%) were included compared to when 10 or fewer observations were included (14.6%) in the analysis. Across all numbers of individuals, the confidence in estimates of the number of communities exceeded 50% only when 20 or more observations per individual (of the 40 possible) were available and was at or near 0 with less than 10 observations for all numbers of individuals (Table 1).

**Table 1:** All sampling regimes (number of individuals and number of observations per individual) where there was sufficient evidence to detect community structure in at least half of all 100 replicates.

Number of Individuals	Number of Observations	Evidence for Community Structure (p<0.05)	Acceptable R_com_	Correct # of Communities
5	40	50	25	6
10	20	60	56	67
10	30	81	59	55
10	40	95	55	44
15	15	62	77	39
15	20	80	68	53
15	30	96	74	67
15	40	100	63	58
20	15	76	72	44
20	20	94	72	49
20	30	99	78	65
20	40	100	59	71
25	10	54	72	2
25	15	88	66	35
25	20	99	78	44
25	30	100	74	68
25	40	100	58	70
35	10	77	58	0
35	15	98	62	29
35	20	100	79	50
35	30	100	84	61
35	40	100	61	68
50	10	95	33	6
50	15	100	79	31
50	20	100	91	43
50	30	100	75	62
50	40	100	52	68
75	10	100	40	3
75	15	100	91	25
75	20	100	93	52
75	30	100	83	66
75	40	100	57	85
99	10	100	46	3
99	15	100	98	24
99	20	100	97	54
99	30	100	96	89

Sufficient evidence for community structure was based on a p < 0.05 when comparing values of Q in sample networks to that of randomized networks. The number of replicates (0–100) with a community assortativity (R_com_) value considered acceptable at greater than the minimum threshold of 0.5 but less than the ‘observed’ network (0.858) are reported and the number of replicates (0–100) that correctly estimated the presence of 3 communities.

**Fig 2:**
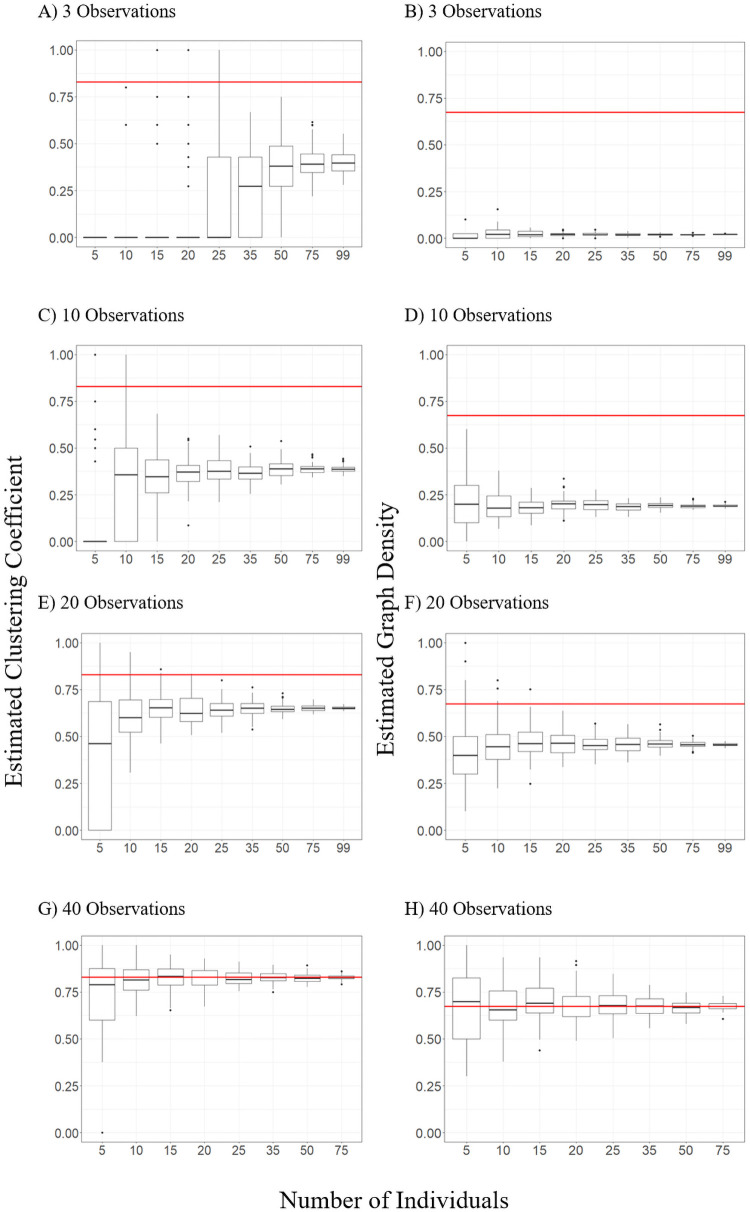
Influence of number of individuals and number of observations per individual on estimates of network clustering coefficient (panels A, C, E, and G) and graph density (panels B, D, F, and H). Boxes represent the 25^th^ to 75^th^ percentile of values with the center line representing the median value. The horizontal red line indicates observed values of graph density (0.675) and clustering coefficient (0.828) based from the ‘observed’ network. Full figures for each measure with all tested numbers of observations per individual can be found in S1 and S2 Figs.

The absolute difference in graph density estimates between the ‘observed’ and sample networks was significantly correlated (R^2^ = 0.797, p < 0.001) with the number of observations per individual (β = -0.108, SE = 0.003, p < 0.001), the number of individuals (β = 0.009, SE = 0.002, p < 0.001), and the interaction between these terms (β = -0.008, SE = 8.987 x 10^−4^, p < 0.001) in the log transformed model. Graph density estimates increased towards the true value of 0.675 with increasing numbers of observations per individual while variance in estimates decreased with more individuals included (Fig 2B, 2D, 2F and 2H). Clustering coefficient changed similarly, with the absolute difference between ‘observed’ and sample estimates related to the number of observations per individual (β = -0.021, SE = 2.467 x 10^−4^, p < 0.001), the number of individuals (β = -0.003, SE = 1.037 x 10^−4^, p < 0.001), and the interaction between these terms (β = 5.604 x 10^−5^, SE = 5.730 x 10^−6^, p < 0.001) in a linear model (R^2^ = 0.690, p < 0.001). Mean estimates of clustering coefficient generally increased similarly with more observations per individual, regardless of the number of individuals, while increasing the number of individuals reduced the variance in clustering coefficient estimates (Fig 2A, 2C, 2E and 2G).

### Relationship between sampling regime and evidence for community structure

**Fig 3:**
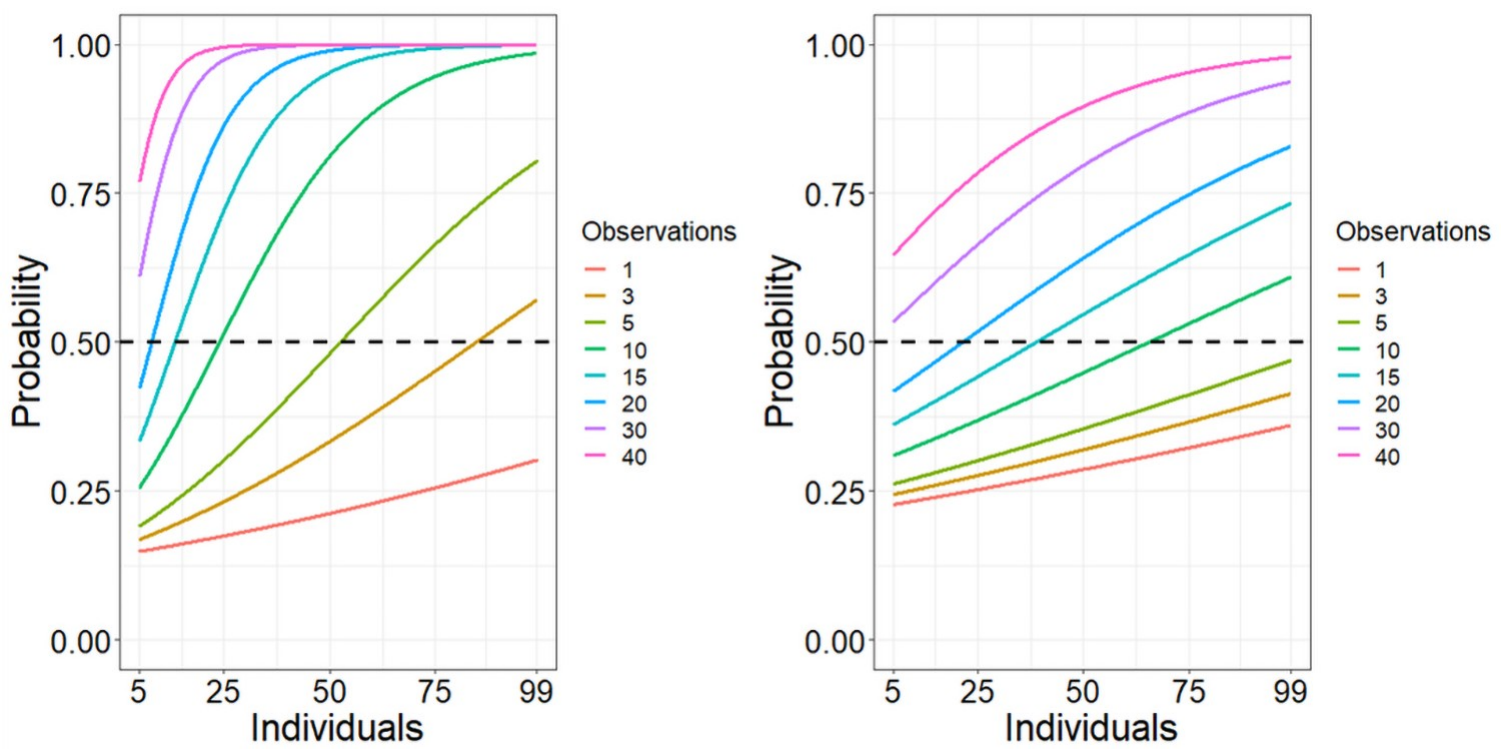
Probability of detecting community structure (A) and confident assortment of individuals (B). Detection of communities (A) is based on the p-value of modularity (Q; p < 0.05) and confident assortment of individuals (B) is based on obtaining a community assortativity coefficient (R_com_) value that indicates a reasonable ability to assort individuals into communities (≥ 0.5) without overestimating this ability based on the ‘observed’ network (≤ 0.858).

Analysis of the p-values of Q indicated a significant increase in the probability of correctly concluding the presence of community structure with additional individuals (β = 0.004, SE = 0.002 p = 0.010) and observations (β = 0.050, SE = 0.005, p < 0.001), and an interactive effect between these variables (β 0.005, SE = 2.709 x 10^−4^, p < 0.001). With < 10 observations per individual, the probability of correctly concluding the presence of community structure did not exceed 75% even with all individuals included. With ≥ 10 observations per individual, the probability of detecting community structure exceeded 50% with < 30 individuals sampled (Fig 3A). At least 10 observations per individual were required to detect the presence of community structure in at least 50% of replicates using the p value of Q (Table 1).

### Relationship between sampling regime and community assignment

**Fig 4:**
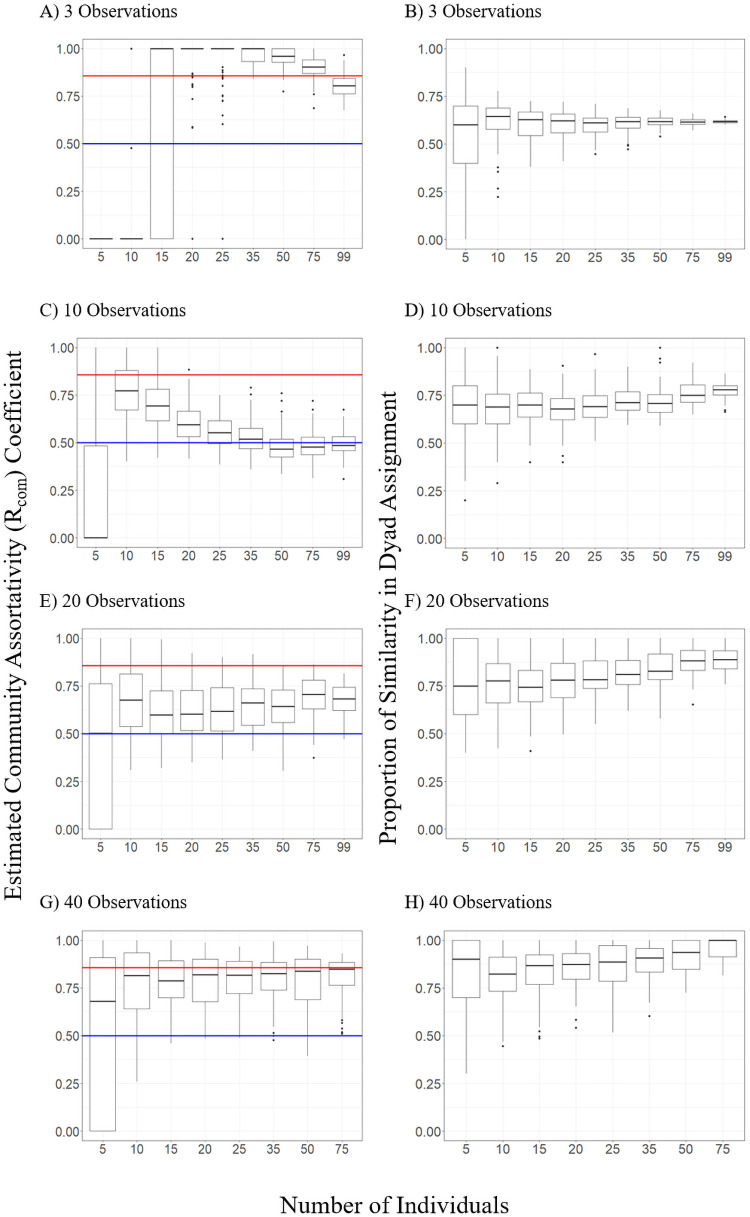
Distribution of community assortativity coefficients (R_com_; panels A, C, E, and G) and proportion of correctly assigned dyads (panels B, D, F, and H) as a factor of the number of individuals and the number of observations per individual included in the network. Boxes represent the 25^th^ to 75^th^ percentile of values with the center line representing the median value. In panels A, C, E, and G, the blue line indicates the suggested minimum threshold of R_com_ (0.5) and the red line indicates the R_com_ value of the ‘observed’ network (0.858). Values that fell within this range were deemed acceptable and therefore represent sample networks with reasonable confidence in the assortment of individuals into subgroups. Full figures for each measure with all tested numbers of observations per individual can be found in S3 and S4 Figs.

R_com_ values ranged from 0 to 1 (median = 0.669, SE ± 3.810 x 10^−3^) across all sampling regimes. We observed a significant relationship between the probability of obtaining a R_com_ value indicating reasonable ability to assort individuals into communities (0.5 < R_com_ < 0.858) and the independent variables of number of individuals (β = 0.006, SE = 0.001, p = 0.01) and number of observations per individual (β = 0.043, SE = 0.003, p < 0.001; Fig 3B). The interaction term between the two independent variables was also significant (β = 7.066 x 10^−4^, SE = 9.248 x 10^−5^, p < 0.001). At least 15 observations per individual were needed to obtain a R_com_ value above 0.5 but less than the ‘observed’ network value of 0.858 in at least 50% of replicates (Table 1). The proportion of R_com_ estimates that fell within this range occasionally decreased with more observations per individual due overestimates (Fig 4D). With 10 observations or fewer, R_com_ was overestimated relative to the observed network in 33.0% of replicates. With 15 or more observations per individual, estimates of R_com_ fell within the acceptable range in 68.7% of replicates and overestimated in 16.9% regardless of the number of individuals included (Fig 4A, 4C, 4E and 4G).

The proportion of dyads correctly assigned to communities based on the ‘observed’ network significantly increased with increasing numbers of observations per individual (β = 0.005 x 10^−3^, SE = 1.637 x 10^−4^, p < 0.0001) and was also significantly affected by the interaction between the number of observations per individual and the number of individuals included in the model (β = 7.061 x 10^−5^, SE = 3.803 x 10^−6^, p < 0.0001). The number of individuals was not a significant predictor of the correct assortment of dyads (p = 0.887). As with graph density and clustering coefficient, increases in similarity were seen with more observations per individual, while the inclusion of more individuals reduced the variance in similarity results (Fig 4B, 4D, 4F and 4H).

## Discussion

In most studies of animal behaviour, it is recommended to include as much information as possible to estimate characteristics with the greatest probability of accuracy and to ensure reliable inference. Social network analysis is no exception, particularly when the gambit-of-the-group method is applied to deducing social connections [14]. However, in ecological studies, sampling is often limited by factors such as logistical challenge and cost. We quantified the effects of both the number of individuals and the number of observations per individual on the reliability of metrics that characterize a fission-fusion social network created using the gambit-of-the-group assumption. When looking at other global network metrics including graph density, the number of connections compared to the number of possible connections, and clustering coefficient, a metric based on the connections among triads, discrepancies from the observed network decreased most notably when including more observations per individual, while including more individuals reduced the variance in estimates. Further, the ability to detect community structure, assort individuals into communities, and estimate the number of communities in the network improved with the inclusion of more data but the addition of more individuals and more observations did not have equal impacts on these different estimates. Thus, when designing studies of animal behaviour, there can be considerable trade-offs between these two sampling considerations and the metrics of interest.

Our results are consistent with prior studies based on estimates of individual network position [15,17] that recommended only including individuals for which a minimum number of observations have been recorded, and that adding more individuals cannot make up for a lack of repeated observations of individuals or interactions [31]. We found that the presence of communities and confidence in individual assortment into communities could often be estimated with missing data but, in our dataset, 15 or more observations per individual were needed to consistently achieve an estimate within 2 of the ‘observed’ number of communities present, regardless of the number of individuals present. Additionally, including a larger number of individuals with less than 10 observations per individual greatly increased the average discrepancy between sample estimates and the ‘observed’ network. As these results demonstrate, including many individuals with insufficient information per individual has the potential to result in increased error when estimating group-level metrics. Yet, the use of overly conservative minimum observation thresholds when estimating network properties may reduce our ability to detect weak connections, which can be essential for understanding other aspects of the overall network structure [21]. It is therefore important to balance including as many individuals as possible with ensuring that all included individuals have been sampled sufficiently.

To select a sampling regime with sufficient potential to estimate animal social networks, our results demonstrate the potential error that can occur. We suggest that other studies with sufficiently large data sets perform similar subsampling tests to assess how their estimates respond to missing data and provide some suggestion of how requirements may change based on study objectives. For most studies interested in aspects of global network structure, the priority is to determine whether the network shows evidence of any community structure. In our ‘sample’ populations, conclusions consistent with the ‘observed’ population regarding the presence of community structure could be made with as few as 10 observations and less than 50% of the population sampled, when using Monte Carlo methods to obtain a p-value for Q. The number of observations per individual had a strong impact on the ability to detect community structure, as networks with < 10 observations with any number of individuals were not reliable and could not detect the presence of communities without nearly all individuals being sampled and even then, still demonstrated some uncertainty. These findings support the recommendations of Franks et al. [14], who suggested that when resources are limited, researchers should prioritize sampling fewer individuals more intensely rather than sampling more individuals less intensely and support that network metrics can be robust to missing individuals when enough observations are provided [19]. However, these results do not provide information on whether the estimated number of communities is accurate, or how well the estimated communities assort individuals.

To accurately estimate the number of communities present in this social network, far more information, and therefore a more intense sampling regime, was required than was needed to simply detect that communities were present. Overall, our ability to accurately estimate the number of communities was limited. Success for any particular sampling regime did not exceed 80% with the exception of having 30 observations per individual and 100% of individuals included or 40 observations per individual and 75 of 99 individuals included. A feat that may be unreasonable for many ecological studies. Estimates of the number of communities did generally improve with the inclusion of more individuals or more observations per individual but it was again important that at least 10 observations per individual were included. With 20–30 observations, the correct number of communities was estimated over 50% of the time, even with only 15 individuals included. These findings are again in agreement with the recommendation of Franks et al. [14]to ensure a sufficient number of observations per individual. Further, it has been previously demonstrated that the ability to estimate attributes of animal networks, and therefore the amount of information needed to detect a biological effect, is dependent on the relative effect size of the attribute under study [31]. Given the densely overlapping nature of communities in fission-fusion societies, it is possible that estimates of communities numbers would perform better in less highly connected networks with the same sampling regime [19,32], as community delineation would be more pronounced in these instances.

Silk et al. [17] suggested that global measures of social networks would perform poorly when individuals are missing from a network. With both global estimates of network graph density and clustering coefficient, our results suggest that missing observations had more impact on estimated values than missing individuals. Further, the ability to provide significant evidence for the presence of community structure based on p-values of Q and the modest ability to estimate the number of communities present despite missing individuals, provided sufficient numbers of observations shown here contradicts Silk et al. [17]’s proposition. This may be due to the fact that some global metrics can be greatly influenced by the omission of single individuals that bridge two subgroups or communities [17,33] however those connections are more likely to be missed if only a small number observations of individuals are recorded. Our results suggest that when selecting a social network metric to determine the presence of communities for populations that are under-sampled, Q may be a robust choice in the face of missing information, while measures such as clustering coefficient showed little accuracy with limited numbers of observations, and great uncertainty with limited numbers of individuals. However, further work is needed to ensure that the performance of these metrics is not unique to our specific network structure. Additionally, the performance of global metrics may vary based on the type of sampling method used in a given study, and the definition of edges in a network [20].

Regardless of the number of communities, the ability to consistently assort individuals to communities is useful for understanding network structure and asking questions about mechanisms of group and community formation. Although assignment of individuals to communities can be uncertain, even with only 3 observations per individual and 5 individuals, dyads were generally assigned correctly over 50% of the time. The proportion of similarly assigned dyads increased most obviously with the addition of more observations per individual and was less variable with more individuals included thus showing a relationship similar to that of network graph density and clustering coefficient. R_com_ suitably increased with greater similarity in dyad assignment to communities based on the ‘observed’ network. R_com_ provides an opportunity to estimate the confidence in the assortment of individuals to specific communities [22] when the underlying network is not available for comparison as we have here. Generally, our analysis of R_com_ demonstrated the performance of assigning individuals to communities was more variable than detecting communities via p-values of Q, but more consistent than estimating the exact number of communities within any given sampling regime. There was a tendency to overestimate the R_com_, a similar result to other assortativity measures, such as assortment into groups by sex or other traits of the individuals [14]. It is unsurprising that the R_com_ may be vulnerable to the same issues. Specifically, we found that R_com_ was often overestimated when individuals were observed < 10 times. This may be due to less variation between bootstrap removals of individuals when networks contained fewer observations. As the number of observations increased beyond 20 per individual, we found that R_com_ stabilized, a result which is corroborated by Franks et al. [14] and emphasizes the benefit of increasing the sampling intensity for each individual.

Unlike other studies on the effects of missing information on network estimates, we do not suggest any specific thresholds for estimation of global network metrics as our results may not be reflective of the exact error trends in other study systems. Specifically, our ‘observed’ network has only a small number of communities, and it is expected that with more communities, estimates from subsamples would be more variable, and the ability to assort individuals in these communities would decline. Thus, more subdivided networks may require a larger proportion of the population to be sampled, and/or more observations per individual to appropriately characterize the network community structure. In addition, the difference in average strength of connections within and among communities may be more variable in other networks. In networks where communities are less connected, such as those of other bat species, *Thyroptera tricolor* [34], fewer observations per individual may be appropriate to delineate communities. Important considerations for differences in the rate of interactions and variability in network structure will also impact sampling protocols [20]. Finally, our ‘observed’ network does not contain all individuals and interactions that occur in the population, and thus it is unknown how well our ‘observed’ network represents the true, underlying population of bats in our study area. This topic would benefit from further study on how global network estimates preform when the underlying population is known with more certainty, for example, using captive populations that are continuously monitored. As our network serves as only an example of how estimates of global network metrics may vary in a specific type of fission-fusion system, we recommend that, as these types of larger datasets become more readily available, researchers continue to assess how subsampling and therefore missing data influences network estimates. There may be additional benefits to testing how community detection, R_com_, the estimated number of communities, and accuracy of assignment to communities vary with network size, as the effect of sampling effort on the accuracy of other network measures has been found previously to vary with overall network size [17]. Given that our ‘observed’ network of 99 tagged individuals was much smaller than what is expected in the true population, and in some other social networks assessed to date (exceeding 1000 individuals [3]), predictive ability may be higher in actual bat social networks when the same proportions of the population are observed. Another potential avenue of future research is to assess how R_com_ can be adapted for less stable animal social societies, including those of bats. It is likely that increasing connectivity among communities despite high densities of connections within subgroups, as is typical in fission-fusion societies, negatively influences our ability to correctly assort individuals to a subgroup. A modification of R_com_ has been suggested for instances where an individual is a part of multiple communities simultaneously [35], and perhaps a similar modification is needed for societies displaying fission-fusion dynamics. Further study is necessary to determine how both network size and structure can influence the consequences of sampling and data filtering prior to generating network models based on a portion of the overall population [17,19].

The temporal extent of data collection also has the potential to influence social network structure and assignment of individuals to communities. Specifically, our data were collected over a relatively long period of time (131 days) representing the full range of dates when at least one animal was observed in the study area. This results in the multiple observations of an individual not necessarily occurring on the same days as observations for other individuals. Although it is likely that days where individuals were not observed occurred when individuals were not present in the system and therefore not associating with the observed animals. Always observing individuals synchronously, as is the case in many animal network study systems, is expected to improve network estimates and possibly result in fewer observations per individual being required to achieve comparable levels of reliability [20]. A shorter, more heavily sampled time period should provide increased confidence in R_com_ and estimates of the number of communities but, shorter time-periods represent only a snapshot of the social environment. Networks are inherently dynamic systems and inferences across too narrow a temporal window may overestimate the strength of relationships between individuals and communities or miss important transient connections [36–39]. Further, obtaining 20 observations but over a very long time-period would likely be insufficient as more samples would be needed to appropriately reflect such a dynamic system throughout the entire study period. Thus, when designing studies, researchers must carefully consider the temporal period of network formation that is biologically relevant for their study species (e.g. reproductive period or breeding season), and the expected rate of interactions or behaviour of interest, and select an appropriate sampling method and frequency for the designated period [20].

## Conclusions

Overall, our study suggests that global metrics, including the detection of community structure and appropriate assortment of individuals into communities, can be robust to missing information while estimating the exact number of communities is less reliable. When designing a study, results in our system support the suggestion of Franks et al. [14] to prioritize sampling a smaller part of the population with greater intensity when resources are limited, rather than more of the population at lower intensity. For some measures, accuracy to the true value improves with more observations per individual, while variation in estimates decreases with more individuals included. Generally, we recommend that researchers include as many individuals and observations as is feasible, but we urge caution when including individuals with limited numbers of observations. To best design sampling protocol, researchers must consider their study system, sampling logistics, decide which measures are most important, and determine what level of reliability is desired. The levels of reliability that we present here reflect that of a highly connected, fission-fusion group and results may differ for different network sizes and characteristics [19]. Our results represent a clear example of the uncertainty in global metric estimates that may exist in samples of animal social networks and continued work is needed to determine how the effects of missing information vary with different types of animal societies. As large datasets becoming increasingly available, and when it is uncertain whether enough individuals or observations have been collected, we demonstrate a benefit to subsampling and repeatedly calculating network metrics, as we have here, to determine if estimates stabilize as the full available sample size is approached.

## Supporting information

S1 Fig Influence of number of individuals and number of observations per individual on estimates of clustering coefficient (all observation levels). Horizonal red line represents the value of the ‘observed’ network at 0.828.
(TIF)

S2 Fig Influence of number of individuals and number of observations per individual on estimates of graph density (all observation levels). Horizontal red line represents the value of the ‘observed’ network at 0.675.
(TIF)

S3 Fig Influence of number of individuals and number of observations per individual on estimates of community assortativity (R_com_; all observation levels). Horizontal blue line represents the threshold value of 0.5 and the horizontal red line indicates the value of the ‘observed’ network at 0.858.
(TIF)

S4 Fig Influence of number of individuals and number of observations per individual on the proportion of similarity in dyad assignment (all observation levels). (TIF)
