# Supplementary figures and images for: Influence of number of individuals and observations per individual on a model of community structure
Source: PLoS One. 2021 Jun 17;16(6):e0252471. doi: 10.1371/journal.pone.0252471 (PMC8211201; doi:10.1371/journal.pone.0252471)

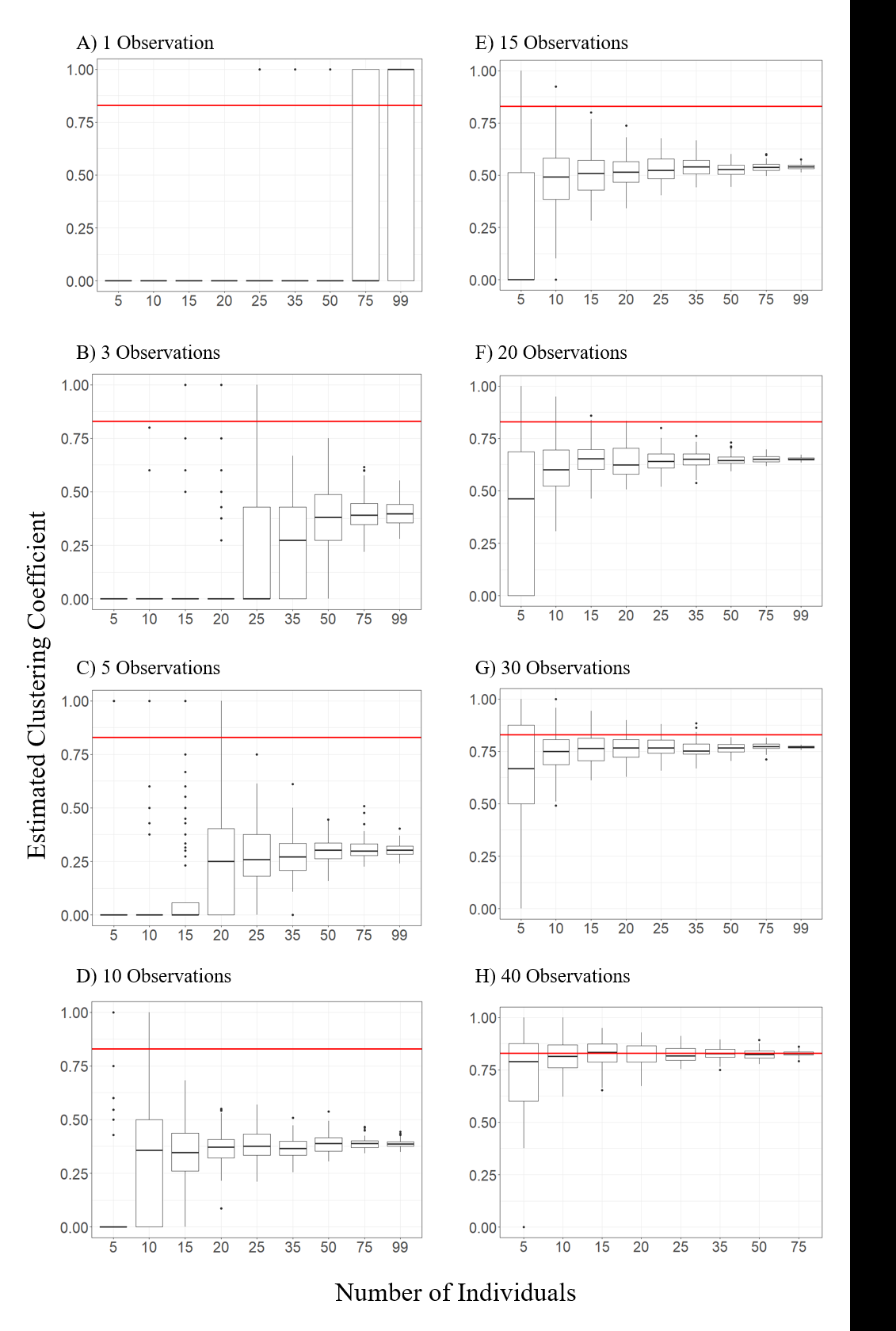

Supplement: S1 Fig — Horizonal red line represents the value of the ‘observed’ network at 0.828. (TIF) [file pone.0252471.s001.tif]

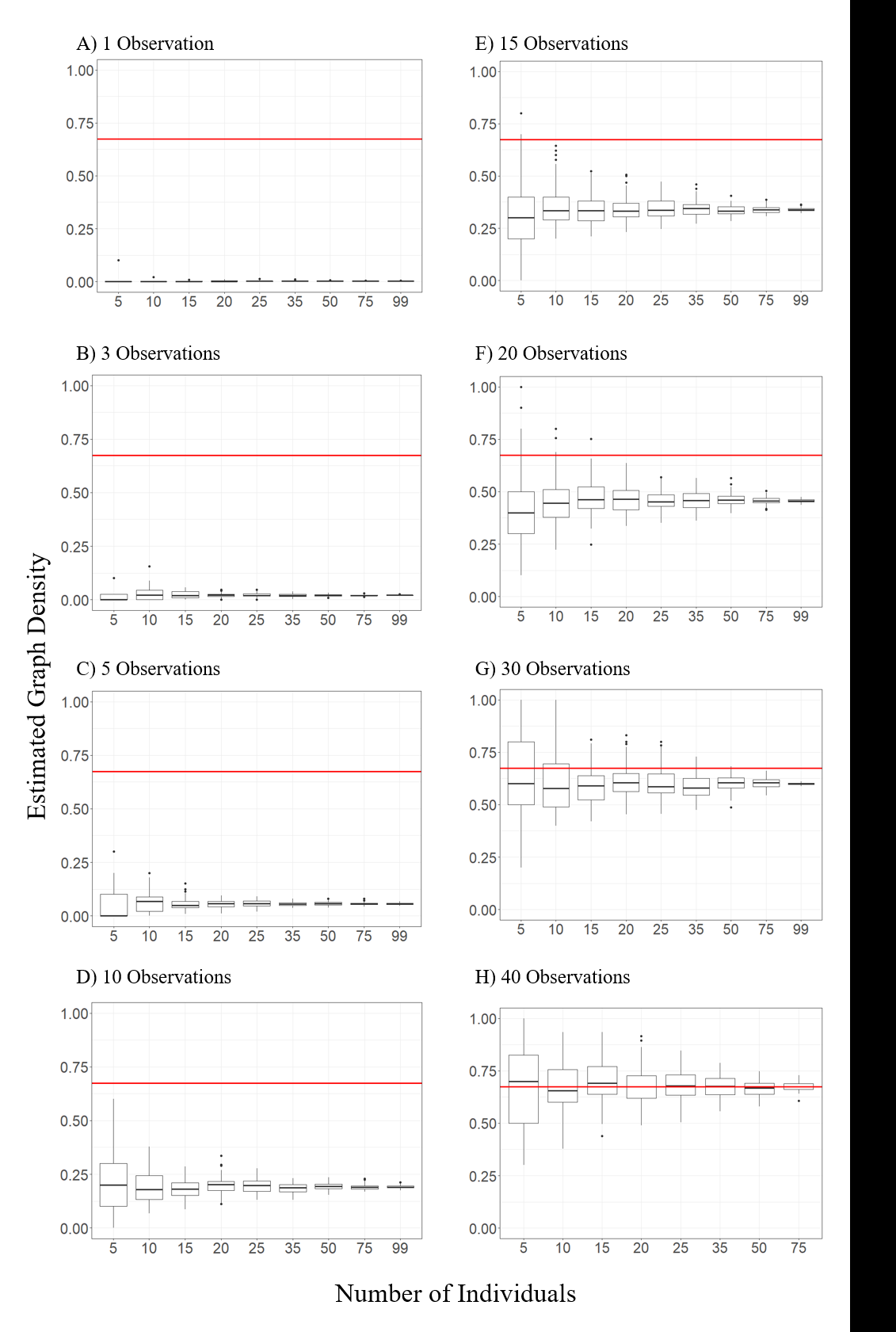

Supplement: S2 Fig — Horizontal red line represents the value of the ‘observed’ network at 0.675. (TIF) [file pone.0252471.s002.tif]

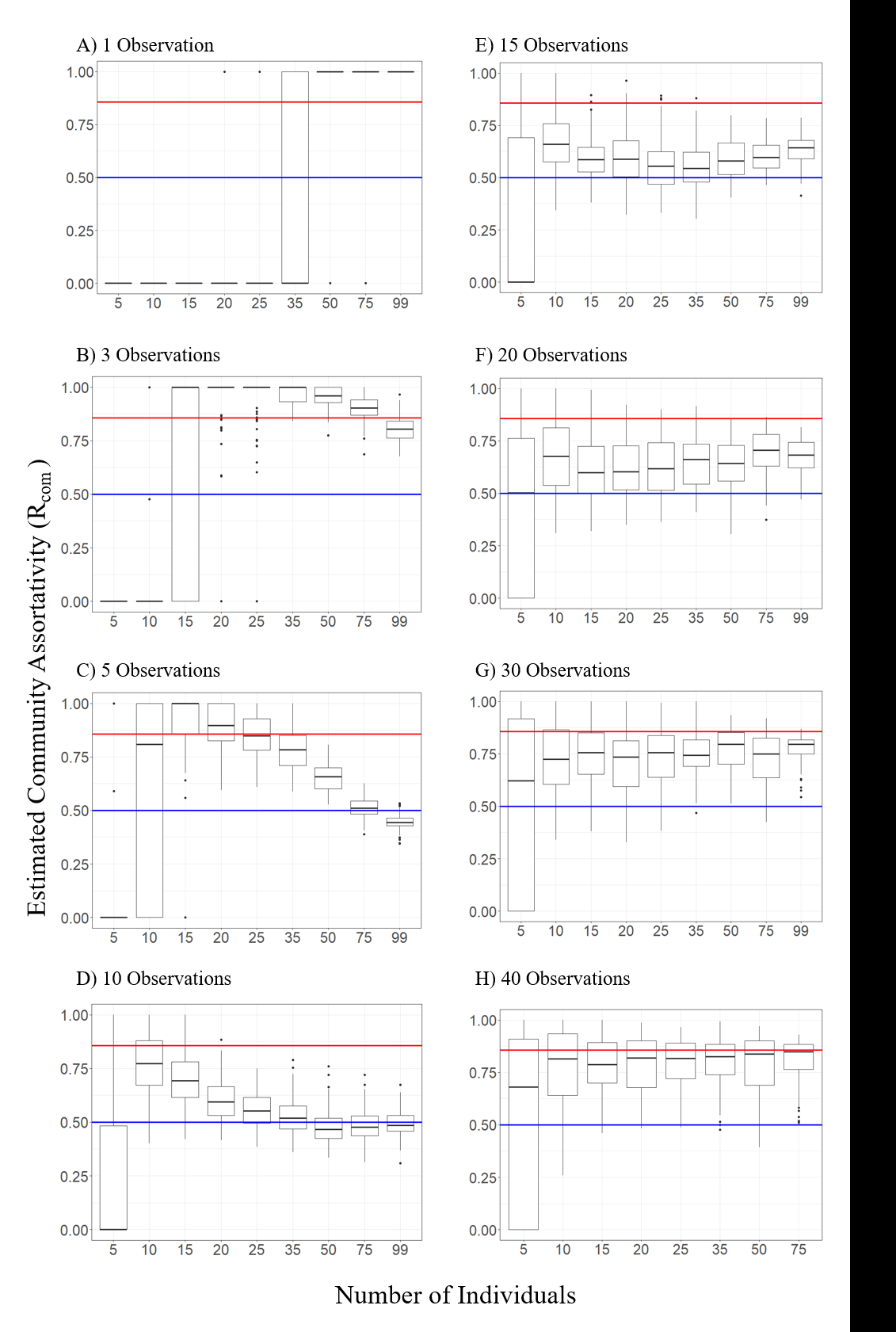

Supplement: S3 Fig — Horizontal blue line represents the threshold value of 0.5 and the horizontal red line indicates the value of the ‘observed’ network at 0.858. (TIF) [file pone.0252471.s003.tif]

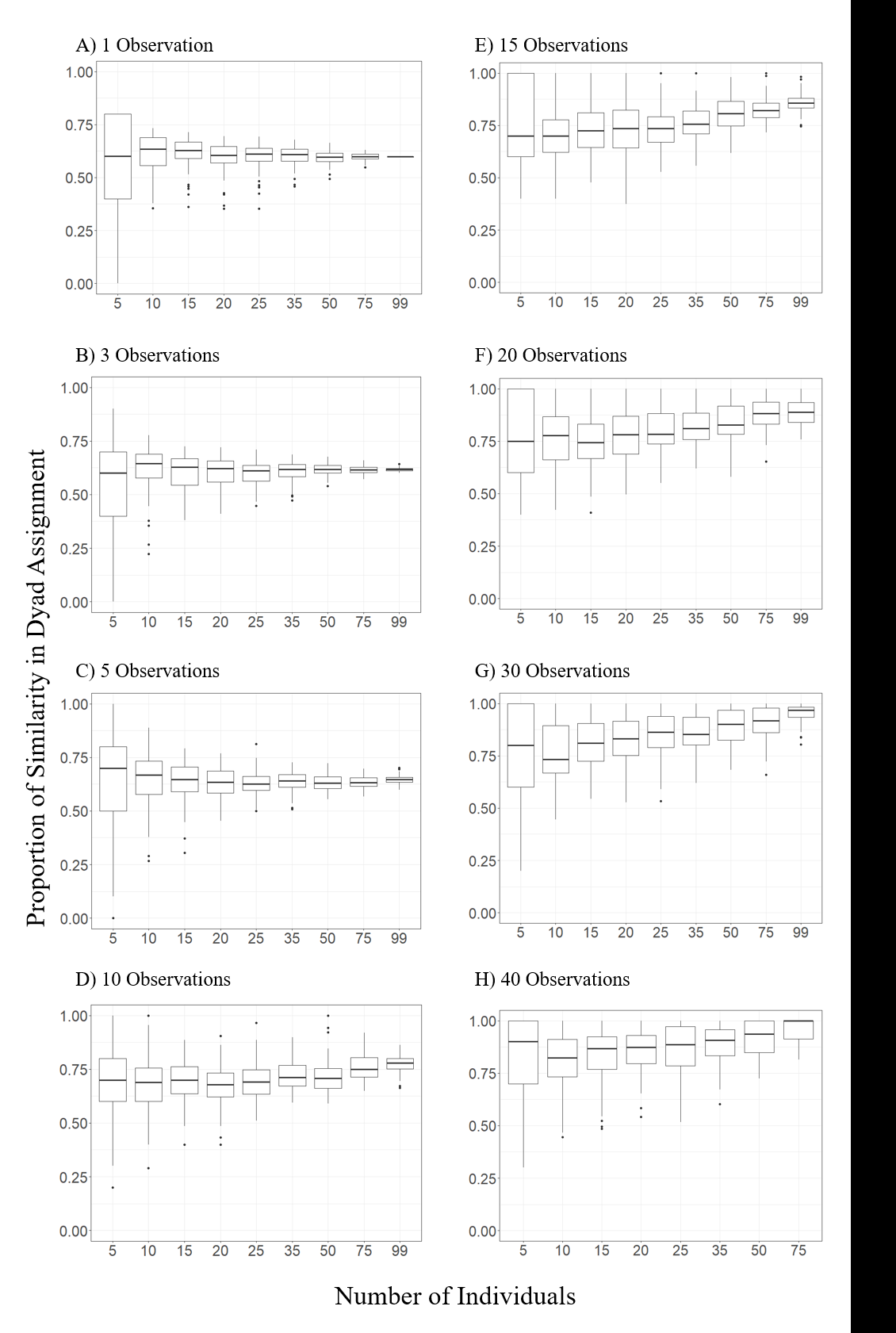

Supplement: S4 Fig — (TIF) [file pone.0252471.s004.tif]
